# Evaluation of breast cancer stem cells in human primary breast carcinoma and their role in aggressive behavior of the disease

**Published:** 2021-09-29

**Authors:** Ninjit Dhanota, Amanjit Bal, Gurpreet Singh, Sunil K. Arora

**Affiliations:** ^1^Department of Immunopathology, Post Graduate Institute of Medical Education and Research, Chandigarh, India; ^2^Department of Radiation Oncology, Weill Cornell Medicine, New York, USA; ^3^Department of Histopathology, Post Graduate Institute of Medical Education and Research, Chandigarh, India; ^4^Department of General Surgery, Post Graduate Institute of Medical Education and Research, Chandigarh, India

**Keywords:** breast cancer stem cell, tumor microenvironment, primary human breast cancer, epithelial to mesenchymal transition, metastatic tumors, extracellular matrix

## Abstract

**Background and aim:**

To delineate the underlying molecular mechanisms responsible for the intratumoral enrichment of breast cancer stem cells (BCSCs) in aggressive breast tumors, we evaluated the frequency and characteristics of BCSCs within the tumor tissue in primary human breast carcinomas. We assessed the expression profiles of various genes in cancer cells (CC) and stromal cells (SC) from these tumors to delineate the role played by the cellular niche in de novo origin or expansion of intra-tumoral cancer stem cells (CSC).

**Method:**

The study included primary tumor and adjacent normal breast tissue specimens from chemotherapy-naïve breast carcinoma patients. The BCSCs, identified as Lin^-^CD44^+^CD24^-^ and aldehyde dehydrogenase 1 A1 positive, were enumerated. The flow-cytometrically sorted stromal, and CC were processed for gene expression profiling using a custom-designed polymerase chain reaction array of genes known to facilitate disease progression.

**Results:**

The frequency of BCSCs within the tumor mass correlated significantly with histopathological and molecular grades of tumors, indicating a direct relationship of BCSC with the aggressive behavior of breast cancer. Further, a significantly increased expression of the genes associated with growth factors, cytokines and matricellular proteins in tumors were found in high BCSCs compared to Lo-BCSC tumors, suggesting the possible contribution of stromal and CC in an intratumoral expansion of CSCs. Similarly, a significant upregulation of genes associated with hypoxia and angiogenesis in Hi-BCSCs tumors further supported the role of a hypoxic environment.

**Conclusion:**

Overall, the findings suggest the molecular crosstalk between SC and CC potentially (directly or indirectly) contributes to the expansion of CSC.

**Relevance for patients:**

The current study highlights the importance of CSC as a potential future predictive/prognostic marker for aggressive breast cancer. The present study predicts the potential risk stratification based on the frequency of BCSCs in primary breast tumors and existing prognostic factors.

## 1. Introduction

The progression of breast cancer is highly unpredictable and dependent on various factors. Advancements in early diagnosis have largely declined the overall mortality rate of patients with breast cancer. Yet, the long-term survival rate with metastatic and recurrent tumors has not improved significantly over the past several decades [1-3]. Accumulating evidence indicates a small population of drug-resistant tumor cells known as “Cancer Stem cells (CSCs),” showing increased metastatic/tumorigenic and stemness potential could be one of the root causes of relapse [4,5]. Although the exact involvement of CSCs in recurrence and metastasis is not well established yet, many putative molecular markers/factors are under surveillance for their possible association [6,7]. It has become interestingly clear that the interactions between tumor cells and stromal cells (SC) play a significant role in the establishment and progression of tumors as well as the expansion and survival of intra-tumoral CSCs [8]. Resistance to anti-cancer chemotherapies commonly involves four fundamental mechanisms; (i) the over-expression of drug transporters/efflux pump, (ii) the manipulation of apoptosis and senescence pathways by cancer cells (CC), (iii) the mechanical or stochastic factors, and (iv) the presence of CSCs [9-11]. After initial therapy, the pan-resistance can be due to the remnant CC, making it far more aggressive and typically unresponsive to any treatment [6].

On the other hand, many reports demonstrate the dynamicity of cells to transform across a spectrum of epithelial and mesenchymal states, as opposed to undergoing a one-way Epithelial to Mesenchymal Plasticity [7,12-15], and the transformed cells have the same phenotypic markers as of CSCs. Besides, the cancer-associated fibroblasts, endothelial cells, and immune cells have also been shown to be associated with secretion of various factors that render the cancer more aggressive, possibly through induction of morphogenetic process called Epithelial to Mesenchymal transition (EMT) [16]. Mechanisms like EMT are well reported for inducing the generation of CSCs/breast CSC (BCSCs) (also known as induced CSC; iCSCs/iBCSCs) through different signaling pathways [17-19]. Subsequently, iCSCs/iBCSCs show enhanced expression of genes related to invasion, migration, metastasis, and chemoresistance [20]. The interaction between CC and SC could initiate a complex signaling cascade, which may be helping in the enrichment of CSCs in the tumor [21].

The critical analysis of the role played by BCSCs in breast cancer metastasis is mainly conceptual and speculative, with a lack of data defining the role of BCSCs in the aggressiveness and progression of breast cancer. We hypothesized that the aggressiveness of breast cancer could be directly related to the enrichment of BCSCs in the tumor microenvironment. Further, we investigated the correlation of metastatic disease with the frequency of BCSCs. Thus, it becomes essential to assess the association/role of various factors released by tumor stroma and the associated mechanisms that could be helping in the enrichment of CSCs in breast cancer. On analysis of the gene expression data, we observed that multiple factors released by the SC seem to significantly influence the intensity and frequency of signals responsible for the expansion of BCSCs.

## 2. Materials and Methods

### 2.1. Study design

Female patients aged 18 – 70 years undergoing mastectomy/lumpectomy (as part of surgical management of breast carcinoma) were recruited in the study. Female patients exposed to chemotherapy and male breast carcinoma cases were excluded from the study. Surgically resected tumor specimens from 100 cases of breast carcinoma were included in the present study (see section 1.1 Specimen Collection of the Supplementary File).

### 2.2. Identification of BCSCs and sorting of CC and SC

Cells were stained with monoclonal antibody conjugates: Lin-FITC, CD44 - PE, and CD24 -APC-H7 fluorochromes (BD Biosciences, USA) to identify CC, SC, and BCSCs by flow-cytometry (see section 1. **Supplementary Materials and Methods of the Supplementary File**). Cell populations based on the phenotypic markers: BCSCs as Lin^-^CD44^+^CD24^-^ cells, breast CC as Lin^-^CD44^+^CD24^+^ and Lin^-^CD44^-^CD24^+^, and SC identified as Lin^-^CD44^-^CD24^-^ and Lin^+^CD44^-^CD24^-^ were analyzed using FACS Diva software (BD Biosciences, USA) and sorted in a flow cytometer sorter (FACS Aria II, BD USA).

In addition, single-cell suspensions obtained from tissue specimens at different tissue intervals from tumors were stained with Lin-FITC, CD44-PE, and CD24-APC H7, CXCR4-APC at 37°C for 15 min in a water bath. Cells were acquired on a flow cytometer (FACS Aria II, BD USA), and populations were analyzed using FACS Diva software (BD Biosciences). BCSCs showing CXCR4 expression were gated and analyzed.

### 2.3. ALDH1A1 expression and scoring

The paraffin sections from all tumors and adjacent normal tissues were stained for ALDHA1 expression by immunohistochemistry (IHC). All staining runs accompanied the appropriate control slides (normal human liver sections). ALDH1A1 staining was also performed on non-metastatic/metastatic lymph node sections of 12 breast carcinoma patients. A pathologist scanned all the stained slides in a blinded manner. (see section 1. Supplementary Materials and Methods of the Supplementary File).

### 2.4. Real-time quantitative polymerase chain reaction (PCR) for gene expression and pathway analysis

The tumor specimens were categorized into two groups irrespective of histopathological grading: Hi-BCSCs tumors (with >5% of BCSCs) and Lo-BCSCs tumors (with <5% of BCSCs) for gene expression profiling of CC and SC sorted from these tumor tissue specimens. The differential expression profile of various genes for stromal factors in sorted populations of CC and SC from the tumor and adjacent tissues were evaluated using a custom-designed PCR array (Supplementary Material). The PCR array included 44 genes related to hypoxia, EMT, growth factors, cytokines, and stromal factors, selected based on their roles in various pathways leading to expansion/origin of CSC (Tables S1-S3). The protein-protein interactions and subsequent biological pathways affected by the differentially expressed genes among various study groups were analyzed using KEGG (http://www.genome.jp/kegg/pathway.html); Reactome (http://www.reactome.org/) and String 9.1 (http://string-db.org/) databases.

### 2.5. Statistical analysis

Discrete categorical data were represented in the form of either an absolute number/percentage or mean ± SE. Continuous data were described either in mean ± SD or in the form of median and interquartile range. Mann–Whitney U test was used for statistical analysis of skewed continuous variables. An Independent t-test was applied to compare normally distributed data of two groups. Data of more than two groups were compared using one-way ANOVA. To evaluate the correlation between different variables, the Spearman correlation test was applied. p-value < 0.05 was considered statistically significant. The analysis was done with the help of GraphPad Prism 5 Version 5.03.

## 3. Results

The present study was conducted in a prospective manner that included treatment naïve cases of breast cancer in female patients (Table 1).

**Table 1:** Clinicopathological characteristics of study subjects (n = 100)

Variable	Number of patients
Total	100
Mean age (range)	51 (26 – 82 years)
Mean tumor size (range)	3.094 (1 – 6 cm)
pT1 (≤2 cm)	26 (28.5%)
pT2 (>2 – <5 cm)	61 (67.0%)
pT3 (≥5 cm)	4 (4.4%)
Axillary lymph nodal status	
Positive (%)	43 (46.2%)
Negative (%)	48 (52.7%)
Pathological Grade (%)	
Grade 1	09 (9.6%)
Grade 2	28 (30.1%)
Grade 3	56 (60.2%)
Breast cancer molecular subtype (%)	
ER^+^/PR^+^	38 (51.3%)
Triple positive	06 (8.1%)
VHER 2^ +^	09 (12.2%)
Triple-negative	21 (28.3%)
Ki67 index:	
≥14%	17 (23.3%)
<14%	56 (76.7%)
Type of surgery	
Mastectomy	79
Lumpectomy	21

The table represents the clinical and histopathological characteristics of 100 treatment naïve breast carcinoma cases. Females presenting with breast carcinoma who were advised mastectomy/lumpectomy, within the age group of 18 – 70 years, were included in the present study at Post Graduate Institute of Medical Education and Research, Chandigarh, India.

### 3.1. Increased frequency of BCSCs correlates with aggressive behavior of breast cancer

**Figure 1:**
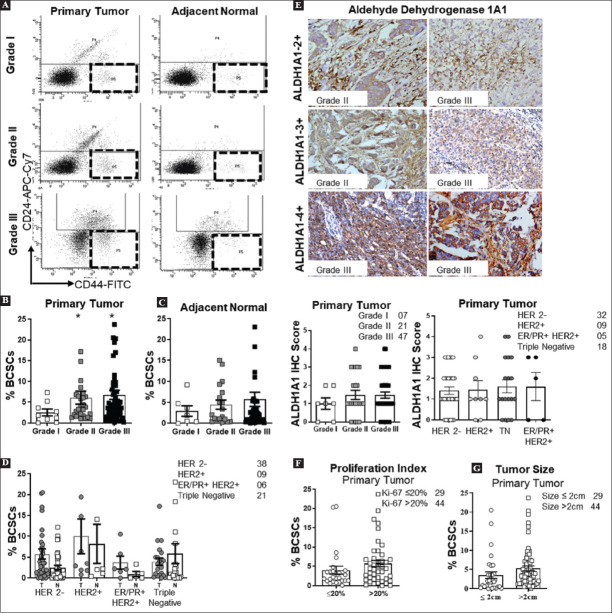
Frequency of BCSCs in tumor and adjacent normal breast tissues of primary breast carcinoma in clinically and pathologically defined aggressive disease setting (A). Flow cytograms representing comparative percentages of BCSCs in various histological grades in tumor (Grade I n = 9; Grade II n = 28; Grade III n = 56) and adjacent normal tissues (Grade I n = 7; Grade II n = 19; Grade III n = 49). Quantification of percentage of BCSCs by flow cytometry (Lin- CD44+ CD24-) in various histopathological grades (B). Primary Tumors (Grade II [p = 0.0369], Grade III [p = 0.032] vs. Grade I) (C) Adjacent Normal tissues. (D) Quantification of percentage of BCSCs in various molecular categories in tumor and adjacent normal tissue (ER/PR+ HER2- n = 38; ER/PR- HER2+ n = 9; ER/PR+ HER2+ n = 6; ER/PR- HER2– n = 21). (E) Representative immunohistochemical staining (40 ×) and quantification of ALDH1A1 in tumor sections in different histological grades. Comparison of IHC scores of ALDH1A1 in various histological grades (bottom panel-left) (Grade I n = 7; Grade II n = 21; Grade III n = 47) and molecular categories (ER/PR+ HER2- n = 32; ER/PR- HER2+ n = 9; ER/PR- HER2- n = 18; ER/PR+ HER2 + n = 5). (bottom panel-right) (F) Differences in percentage of BCSCs in tumors sized ≤2 cm and >2 cm (G) Differences in percentage of BCSCs in Ki-67 ≤20% and >20% tumors. Bars represent Mean, and error bars represent ± SEM. Unless mentioned, statistical comparisons between groups were performed using Kruskal–Wallis and Dunn’s multiple comparisons tests. *p < 0.05, **p < 0.01, ***p < 0.001.

**Figure S1:**
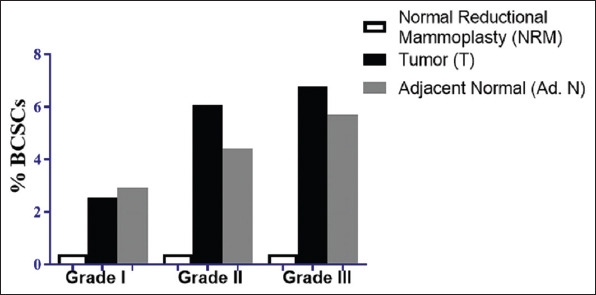
Bar chart showing frequency of BCSCs in different grade tumor tissues compared to normal reductional mammoplasty tissue and adjacent normal tissue

In 2003, Al-Hajj *et al*. identified BCSCs in breast tumors with a phenotype of Lin^-^ CD44^+^ CD24^-^ using flow cytometry [15], which is well accepted now. The frequency of BCSCs (Lin^-^CD44^+^CD24^-^ cells) in tumor samples was expressed as a percentage of total tumor cells by flow cytometry. Although it did not relate well with their molecular categories, a significant increase in BCSCs cell population was observed in high-grade tumor samples (Figure 1A and B). However, it did not relate well with their molecular categories (Figure 1D). We found the adjacent normal tissue also contains BCSC-like cells (Figure 1C and D). The adjacent normal tissue to the tumor was taken as paired control samples in mastectomy samples, but it does not represent/resemble normal mammary tissue (Figure S1). Fifty-five out of seventy-five (73.3%) tissues showed positivity for ALDH1A1 (Figure 1E) by IHC. Stromal positivity was excluded. An increasing trend in ALDH1A1 positivity was seen in histopathological grades (9.1% in Grade I, 23.6% in Grade II, and 67.2% in Grade III tumors). However, the difference did not reach a significant level (Figure 1E and Table 2). However, no statistically significant difference was noted between ALDH1A1 positive and ALDH1A1 negative categories when the tumors were further stratified among four molecular subgroups as Luminal A, Luminal B, TN, and HER2+ (p = 0.528) (Table 2). We found higher ALDH1A1 expression in molecularly aggressive breast cancer consisting of 42.9% of the ALDH1A1 positive group (21 out of 49), suggesting an association of ALDH1A1 with aggressive breast carcinoma.

**Table 2:** Comparison of clinicopathological variables with ALDH1A1 positive or ALDH1A1 negative cases

Clinicopathological parameters	ALDH1A1^+^ CASES (n=26)	ALDH1A1^-^CASES (n=24)	p-value
Mean age (range)±SD	52.5 (26 – 82)*±13.025	53.5 (43 – 70)±8.937	0.796
Mean tumor size±SD	3.32±1.291	2.46±1.117	0.042
Tumor Grade			
I	5 (9.1%)	2 (10%)	0.371
II	13 (23.6%)	8 (40%)	
III	37 (67.3%)	10 (50%)	
Molecular Sub groups			
TP	03	02	0.528
TN	14	04	
HER2^+^	07	02	
HER2^-^	25	7	
Metastatic Lymph Node			
Present	29	29	0.4035
Absent	11	7	
Median Lin^-^CD44^+^CD24^-^	3.7 (1.40 – 9.15)	4.2 (0.67 – 14.52)	0.927

The frequency of BCSCs does not correlate with clinicopathological parameters; the Proliferation Index Ki-67 and Tumor size (Figure 1F and G and Table 2). Although, ALDH1A1 expression was associated with tumor size (p = 0.042) (Table 2).

Clinicopathological characteristics were compared between the ALDH1A1 positive and ALDH1A1 negative groups. Significant differences were found in tumor sizes between the ALDH1A1 positive and ALDH1A1 negative groups.

### 3.2. BCSCs in metastatic lymph nodes and normal adjacent tissue indicate their role in invasiveness and metastasis

**Figure 2:**
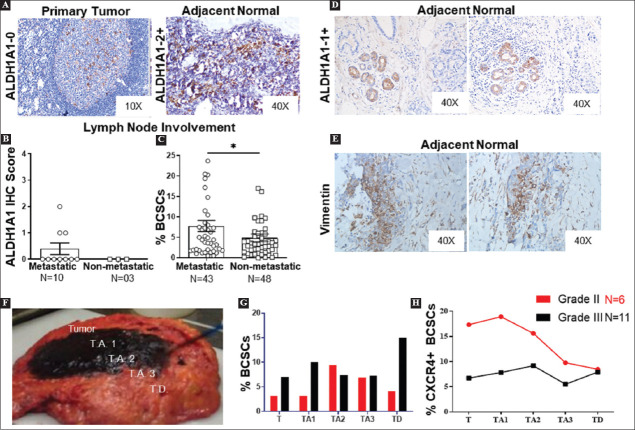
Invasive behavior of BCSCs and their involvement in breast cancer metastasis (A). Representative immunohistochemical stained micrographs of ALDH1A1 staining in metastatic/non-metastatic lymph nodes. (B) Bar graph representing the percentage of BCSCs in Metastatic LN tumors (n = 43) and non-metastatic LN tumors (n = 48) by flow cytometry (C) ALDH1A1 staining on lymph node sections in metastatic (n = 10)/non-metastatic (n = 3) lymph node category (D) Presence of ALDH1A1+ cells within well-arranged histologically normal mammary ducts in tumor vicinity (n = 3) (E) Immunohistochemical staining for vimentin (n = 3) on adjacent normal sections near tumor vicinity (F) Representative mastectomy specimen serially dissected by histologist to obtain following tissues: primary tumor (T), T.A. 1 (Tumor Adjacent 1; 3 mm from T); T.A. 2 (Tumor Adjacent 2; 1 cm from T); T.A. 3 (Tumor Adjacent 3; 2 cm from T); T.D. (Tumor Distant; 4 cm from T) (G) Quantification of the percentage of BCSCs at the primary tumor site and different tissue intervals in grade II (n = 6) and grade III (n = 11). (H) Line plot representing the distribution of CXCR4 expressing BCSCs at different tissue levels (Grade II n = 6; Grade III n = 11). For all data, bars indicate means, and error bars indicate ±SEMs. **p < 0.01, ****p < 0.0001.

Lymph node (LN) metastasis is believably the initial step of distant metastasis and is directly related to a poor prognosis. A positive correlation between LN metastasis and number of BCSCs was observed (p = 0.0218 Spearmen Correlation r = 0.2120). We found 30% lymph nodes with metastatic deposits showing ALDH1A1 positivity; however, non-metastatic LNs had no ALDH1A1 positive cells (n = 3) (Figure 2A and B). The ALDH1A1 positivity was significantly higher in the metastatic lymph node group (69%) compared to the non-metastatic lymph node group (48%), although the difference was not statistically significant (Figure 2C). The presence of BCSC-like cells in adjacent normal tissue to tumors was also observed (Figure 2D). No such cells were found in normal breast reduction mammoplasties (data not included), suggesting dynamic changes in the tumor and its surrounding tumor microenvironment.

In addition, vimentin expression in 5 – 10% tumor cells suggests that possibly these cells have been transformed to mesenchymal cell type (Figure 2E). To evaluate the possible presence of BCSCs in surrounding tissue to the primary tumor, we assessed the expression of CXCR4 on BCSCs at different tissue intervals from the primary tumor site (Figure 2F). We observed percentage of CXCR4 expressing BCSCs followed an increasing trend with the distance from the primary tumor site (Figure 2G and H).

### 3.3. Differential gene expression profiling in CC and SC from Hi-BCSCs tumors and Lo-BCSCs tumors

**Figure 3:**
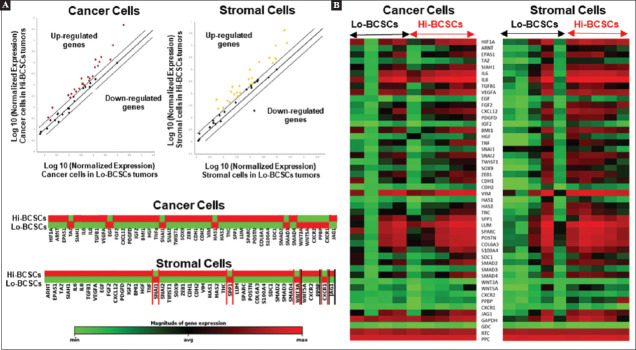
Differential gene expression profile of cancer cells and stromal cells in tumors with high BCSCs versus low BCSCs tumors (A) Scatterplot showing the differentially expressed genes in cancer cells and stromal cells in Hi-BCSCs tumors (16.68%) versus Lo-BCSCs tumors (2.52%). Cluster diagram showing average gene expression for individual genes in cancer cells (upper) and stromal cells (lower) in Hi-BCSCs tumors versus Lo-BCSCs tumors. (B) Heat map showing differential gene expression of selected gene sets in cancer cells and stromal cells isolated from Hi-BCSCs tumors and Lo-BCSCs tumors. Low gene expression is represented by green color, and high gene expression is symbolized by red color in the test versus control. Statistical comparisons between groups were performed, using Kruskal–Wallis and Dunn’s multiple comparisons tests.

Transcriptomic analysis using a shortlisted gene panel was performed in flow-cytometrically sorted cancer and SC from 20 tumor samples of different histopathological grades and BCSCs frequencies. The tumors were divided into High BCSCs (Hi-BCSCs: Tumors with >5% BCSCs) and Low BCSCs (Lo-BCSCs: Tumors with <5% BCSCs) category. The mean values of BCSCs in Hi-BCSC and Lo-BCSC tumors were 16.68% and 2.52%, respectively. Further, stromal and CC were sorted from these tumors for RNA analysis. Thirty-nine genes were found to be upregulated in SC and CC, out of which 26 genes showed two-fold up-regulation in Hi-BCSC tumors compared to Lo-BCSC tumors (Figure 3A and B and Table S4A and B). These genes are associated with hypoxia and its affected genes (HIF1A, ARNT, EPAS1, SIAH1, ZEB1, and TAZ), inflammatory cytokines (IL-6, IL-8, TGF-β1, and TNF-α), growth factors (VEGFA, FGF2, PDGFD, and HGF), EMT (TWIST1, SOX9, CDH1, CDH2, and VIM), and matricellular proteins (LUM, COL6A3, HAS2, POSTN, TNC, SPP1, and SPARC).

Among the four downregulated genes in SC (Figure 3A and B), only one gene was significantly (≥2 fold) downregulated in the Hi-BCSC group compared to the Lo-BCSC group. The downregulated genes included the genes involved in EMT (SNAI1; seven-fold), genes of extracellular matrix proteins (HAS1, SPP1) engaged in aggressive behavior of the disease, and a signaling molecule (WNT 3A). Similarly, in CC, downregulated genes are involved in EMT (SNAI1; TAZ), Chemokine receptors (CXCR2), genes of extracellular matrix proteins (HAS1) involved in aggressive behavior of disease and signaling molecules (WNT 3A, WNT5A, SMAD 3).

**Figure 4:**
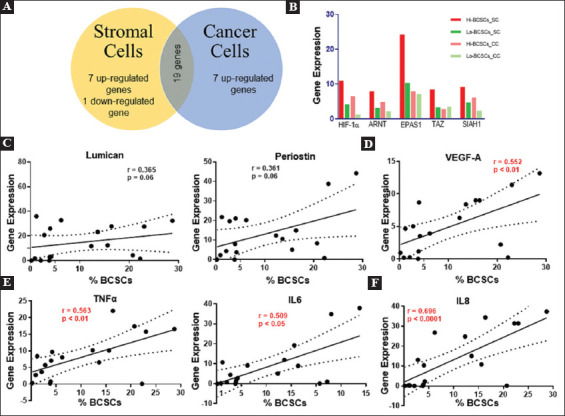
High BCSCs in primary tumor correlates with the inflammatory tumor microenvironment (A). A total of 19 genes were commonly overexpressed by cancer cells and stromal cells, whereas seven genes were exclusively over-expressed by stromal cells and cancer cells. Only one gene, SNAI1, was found to be significantly under-expressed in the stromal cell compartment. (B) Expression profiles of hypoxia-related genes in cancer cells and stromal cells in Hi-BCSC tumors versus Lo-BCSC tumors. (C) Correlation of BCSCs percentage with gene expression of ECM genes: Lumican and Periostin. (D) Correlation of BCSCs percentage with gene expression of VEGFA (E) Correlation of inflammatory cytokines (TNFα, IL6, IL8) with BCSC expansion. The Spearman correlation test evaluated the correlation. p < 0.05 was considered statistically significant.

Deep analysis and scrutiny of gene expression data revealed that among the over-expressed genes in Hi-BCSC tumors, 19 genes were commonly upregulated in stromal and CC. Seven genes were exclusively over-expressed by SC and CC (Figure 4A). Only one gene, SNAI1, was significantly down-regulated in the SC compartment of Hi-BCSC tumors compared to Lo-BCSC tumors (Figure 3A). To evaluate the role of various cellular components known to be part of the BCSC niche, we mainly focused on hypoxia, ECM components, cytokines, chemokines, and growth factors (Figure 4B-E). The analysis revealed a significant positive correlation between the percentage of intratumoral BCSCs with the expression level of VEGFA (Spearman’s p = 0.552, p < 0.05) and IL-6 (Spearman’s p = 0.509, p < 0.05) in the SC (Figure 4C-E) (Table S5).

## 4. Discussion

The failure of existing treatment modalities and residual disease in cancer has been linked to the presence of CSC in previous reports. Some studies report their role in resistance to conventional therapy, while others support their role in relapse and metastasis [22]. Our present study indicates a direct association of aggressive behavior of breast cancer with the frequency of CSCs in the tumor. The unintentional bias introduced due to the large sample size in the Grade 3 tumors category could be a possible limitation. Proposed explanations for the observed increase in the number of CSCs in aggressive tumors could be attributed to many reasons, namely, a) abnormal expression of factors associated with CSC proliferation in the tumor, b) conversion of non-CC to CSCs (de-differentiation) by aberrant/dysregulated signaling pathways, and c) the intensified signals responsible for the intratumoral expansion of BCSCs under the influence of stroma or positive feed-back by BCSCs themselves [23,24]. The specific mechanisms by which CC and surrounding non-CC influence the CSCs’ expansion and formation of primary and metastatic niche sites are currently under investigation. A differentially downregulated expression profile of SNAI1, Wnt ligands in Hi-BCSC versus Lo-BCSC tumors suggests a post-EMT condition. Furthermore, the upregulated genes were associated with hypoxia and inflammation along with concomitant overexpression of growth factors, suggesting a continuous surge of stimuli from SC and CC contributing to the intratumoral expansion of CSCs.

Direct cell-cell interactions between the SC compartment, CC, and CSCs, as well as signaling pathways mediated through the expression and secretion of a range of growth factors and cytokines, play an essential role in the maintenance of the CSC pool within the niche and overall tumor growth [25,26]. As the tumor progresses, the normal stroma undergoes a desmoplastic reaction through drastic changes and expansion [27]. This desmoplastic expansion of stroma results in increased signals coming from activated fibroblasts, myofibroblasts, and inflammatory cells, resulting in ECM remodeling and neovascularization [27]. Observations from our present study support above mentioned notion, as we found a significantly increased expression of growth factors such as VEGFA, FGF2, PDGFD, and HGF that are secreted by SC (fibroblasts, endothelial cells, myofibroblasts, and immune cells) and CC in Hi-BCSC tumors as compared to Lo-BCSC tumors. The increased expression of the S100A4 gene, associated with myofibroblasts, indicates that the SC might help induce migratory properties in CSCs and play a significant role in driving metastasis. In one of the previously reported studies, removing endothelial cells from the CSC niche resulted in a decrease in the CSC numbers, suggesting the dependence of CSCs on various cells of the niche for their maintenance [28,29].

Several experimental, clinical, and epidemiological studies have revealed that chronic inflammation contributes positively to cancer progression [30-32]. Cancer-associated inflammation is mediated by infiltration of leukocytes and secretion of various cytokines such as TNF-α, IL-6, IL-8, TGF-β1, and IL-10 [33,34]. The inflammatory cytokines secreted by a broad range of cells, including immune cells, fibroblasts, and endothelial cells present in the CSC niche [33,34], strongly support the association of cytokines with the maintenance and expansion intratumoral CSC pool [35-37]. A significantly increased expression of IL-6, IL-8, TGF-β1, and TNF-α in SC and CC isolated from primary breast tumors having a high percentage of BCSCs; further supports the significant role of inflammatory tumor environment in the expansion of CSCs.

A close link between EMT and acquisition of CSC-like properties has enabled a greater understanding of the molecular mechanisms underlying the expansion and maintenance of CSCs in tumor masses [17,38,39]. Besides the growth factors such as HGF, VEGFA, EGF, FGF2, and TGF-β1, the hypoxia-related factors include HIF1A, ARNT, EPAS1, TAZ, and SIAH1, and neovascularization are also associated with EMT induction. Our gene expression data reveals that the factors known to regulate the EMT transcription factors such as TWIST1, SOX9, SNAI1, and SNAI2, and mesenchymal markers such as Vimentin and N-cadherin, were all found to be significantly highly expressed in Hi-BCSC tumors as compared to Lo-BCSC tumors, which suggests the significant role played by EMT induction in the expansion of CSCs in the breast cancer. It is in concordance with the earlier reports supporting that induction of EMT helps acquire CSC-like properties during tumor progression [17,18].

CSCs reside in a unique microenvironmental niche that provides significant cues for promoting survival and maintenance [40,41]. The expression of hyaluronan synthase 2 (HAS2) involved in the production of hyaluronan and collagen (COL6A3), being the primary ECM components of the CSC niche, also play a precarious role in CSC enrichment [42]. In our study, these genes were upregulated in Hi-BCSC tumors versus Lo-BCSC tumors, supporting evidence that ECM integrity determines the BCSC expansion. In addition, matricellular proteins such as Lumican (LUM), osteonectin (SPARC), osteopontin (SPP1), tenascin (TNC), and periostin (POSTN) associated with aggressive behavior of the disease, known to induce and formation of the pre-metastatic niche [4,43] were also found to be upregulated in Hi-BCSC tumors, thus suggesting an association of matricellular protein expression with BCSC expansion.

## 5. Conclusions

Our study reveals that the stroma around CSC releases many factors that can promote the growth of BCSCs. Accordingly, we propose, the growth factors and inflammatory cytokines in the tumor microenvironment induce transcription factors, which initiate EMT, thereby facilitating enrichment of CSCs.

## Data Availability

Technical appendix, statistical code, and dataset are available from the first and corresponding author at arora.sunilkumar@pgimer.edu.in.

## Appendix

**ORIGINAL ARTICLE**

Supplementary: Evaluation of breast cancer stem cells in human primary breast carcinoma and their role in aggressive behavior of the disease

**1. Supplementary Materials and Methods**

*1.1. Specimen collection*

Total mastectomy and axillary clearance (TMAC) and lumpectomy specimens containing primary breast tumor and adjacent normal tissue (taken from the farthest distant site of the primary tumor without tampering with the resection limits of tissue) were collected. Specimens collected in DMEM medium supplemented with antibiotics and fetal bovine serum (FBS) were transported to the Molecular Immunology Laboratory aseptically. Samples from reduction mammoplasties and other cosmetic surgeries were collected as normal control.

For studying migration markers on breast cancer stem cells (BCSCs), we collected tissue specimens at different tissue distances from the tumor (T- at the primary tumor site, T.A.1- at 3 mm away from the tumor, T.A.2- at 1 cm away from the tumor, T.A.3- at 2 cm away from the tumor, and T.D.- at 4 cm away from tumor margins) from 17 TMAC cases.

For flow cytometry experiments, fresh specimens were processed for single-cell suspensions. Immunohistochemistry (IHC) and paraffin blocks were prepared from formalin-fixed samples from the primary tumor and adjacent normal tissue.

*1.2. Characterization of BCSC*

1.2.1. Mammospheres forming assay

Sorted BCSCs (Lin^-^ CD44^+^ and CD24^-^) were characterized by mammosphere forming assay. Sorted BCSCs were seeded in a density of 40,000 cells per mL in Mammocult medium supplemented with growth factors (Stemcell Technologies, Canada) in ultra-low attachment condition plates (Corning, USA). Mammosphere formation was observed at alternative days till the 7^th^ day. Primary mammospheres were dissociated with 0.25% trypsin and seeded again for secondary mammosphere formation.

1.2.2. Stemness gene expression profiling

RNA was extracted from the sorted BCSCs population. cDNA was prepared using a Thermo verso cDNA preparation kit (Thermo Fisher Scientific, USA). Expression of classical stemness genes (SOX2, Nanog, OCT4, KlF4, and ABCG2) was determined by end-point evaluation of the transcripts by reverse transcriptase-polymerase chain reaction (RT-PCR).

1.2.3. Identification of BCSCs expressing CXCR4 expression

Single-cell suspensions obtained from tissue specimens at different tissue intervals from tumor were stained with Lin-FITC, CD44-PE, and CD24-APC H7, CXCR4-APC at 37°C for 15 min in a water bath. Cells were acquired on a flow cytometer (FACS Aria II, BD USA), and populations were analyzed using FACS Diva software (BD Biosciences). BCSCs showing CXCR4 expression were gated and analyzed.

*1.3. Identification of BCSC with aldehyde dehydrogenase 1A1 (ALDH1A1) expression by immunohistochemistry (IHC) staining*

The paraffin sections from all tumors and adjacent normal tissues were stained with hematoxylin and eosin (H and E) for histopathological grading. Paraffin sections from the same tissue blocks were deparaffinized in xylene and rehydrated in ethanol for immunohistochemistry (IHC). Antigen retrieval was performed in heat-induced epitope retrieval in citrate buffer (pH 6.0) for 10 min. Endogenous peroxidase activity was blocked by incubation in 1% hydrogen peroxide (H_2_O_2_) for 10 min. Incubation with primary antibody (1:50) was carried out at room temperature (RT) for 1 h. The secondary antibody (HRP Detector and Chromogen; Cell Marque, USA) was applied for 30 min at RT after washing with tris-buffered saline (TBS). Diaminobenzidine (DAB) solution was used for color detection, followed by counterstaining with hematoxylin. All staining runs were accompanied by appropriate control slides (normal human liver sections). We also have performed ALDH1A1 staining on non-metastatic/metastatic lymph node sections of 12 breast carcinoma patients.

The positive to negative cellular profiles ratio was estimated as a percentage of all tumor cells in a slide. The intensity of ALDH1A1 expression was scored in tumor cells only. Stromal positivity (Leucocytes, Macrophages, Adipocytes, Mesenchymal cells present in stroma) was considered negative. Liver sections were used as a positive control for validating the ALDH1A1 staining on tumor sections. A histological score was obtained by counting the positive tumor cells with a score ranging from 0 to 4+. To classify patients into ALDH1A1 (+) and ALDH1A1 (-) groups, ALDH1A1 (+) tumor sections were scored as 4+ (≥50% positive tumor cells), 3+ (≥10% – <50%), 2+ (≥5% - <10%), 1+ (1 – 5%), and 0 (Negative). For the analysis, all 1+, 2+, 3+, and 4+ were considered positive.

*1.4. Custom-designed PCR Array*

The expression profile of various genes for stromal factors in different sorted populations of cells from the tumor and adjacent tissue was evaluated using a custom-designed PCR array. The PCR array included 44 genes related to hypoxia, EMT, growth factors, cytokines, and stromal factors, selected based on their roles in various pathways leading to expansion/origin of cancer stem cells (Tables S1 and S2).

The custom PCR array was designed, and customized plates were received from Qiagen (RT^2^ Custom Profile PCR array Human, Qiagen) (Tables S1-S3). We used RT^2^ SYBR Green Master Mix (Qiagen, Germany) for Real-Time PCR in a 96-well PCR plate format (48 genes, two samples per plate) using Light Cycler 480 II (Roche, Germany). Expression values of various genes were normalized against the house-keeping gene (GAPDH) of the same sample. Other controls such as Positive PCR control (PPC), Genomic DNA contamination (GDC) control, and Reverse Transcriptase Control (RTC) were also used as per recommendations of the manufacturer.

*1.5. Total RNA Extraction*

Total RNA was extracted from sorted cells using TRI Reagent (Sigma-Aldrich, USA). 1-Bromo-3-Chloropropane was added to the sample and vortexed for phase separation. The mixture was allowed to stand at room temperature for 15 min and centrifuged at 12,000×g for 15 min at 2 – 8°C. The aqueous layer was carefully transferred into a new RNase-free microcentrifuge tube and processed using a standard protocol as provided in the RNeasy Mini kit. RNA from the spin column was eluted using 14 μL of RNase-free water by adding directly onto the center of the membrane. The concentration and purity of RNA in the specimen were determined by reading the optical density at 260 nm and 280 nm (Nanodrop 2000).

**Table S1:** List of selected genes based on their involvement in the expansion of CSCs for custom PCR array

No.	Various factors/mechanisms involved in the expansion of CSCs	Genes
1.	Stromal factors/growth factors/cytokines involved in CSC self-renewal, proliferation, maintenance, migration	IL-6, IL-8, TGF-β1, VEGFA, EGF, FGF2, CXCL12, PDGFD, IGF2, BMI1, HGF, TNF-α
2.	Genes related to Hypoxia and EMT- the mechanism involved in CSC expansion	HIF1A, ARNT, EPAS1, TAZ, SIAH1, SNAI1, SNAI2, TWIST1, SOX9, ZEB1, CDH1, CDH2, VIM
3.	Extracellular proteins involved in CSC expansion, self-renewal, maintenance of CSC niche	HAS1, HAS2, TNC, SPP1, LUM, SPARC, POSTN, COL6A3, S100A4, SDC1
4.	Signaling molecules involved in CSC expansion, self-renewal, maintenance	SMAD2, SMAD3, SMAD4, WNT3A, WNT5A, JAG1
5.	Chemokines/chemokine receptors involved in CSC self-renewal, maintenance	CXCR2, CXCR1, PPBP

**Table S2:** Gene symbols and their official full names

Gene symbol	Official full name
HIF1A	Hypoxia Inducible Factor 1, Alpha Subunit
ARNT	Aryl Hydrocarbon Receptor Nuclear Translocator
EPAS1	Endothelial PAS Domain Protein 1
TAZ	Tafazzin
SIAH1	Seven In Absentia Homolog 1
IL-6	Interleukin 6
IL-8	Interleukin 8
TGF-β1	Transforming Growth Factor- Beta 1
VEGFA	Vascular Endothelial Growth Factor A
EGF	Epidermal Growth Factor
FGF2	Fibroblast Growth Factor 2
CXCL12	Chemokine (C-X-C) ligand 12
PDGFD	Platelet Derived Growth Factor D
IGF2	Insulin-Like Growth Factor 2
BMI1	BMI1 Polycomb Ring Finger Oncogene
HGF	Hepatocyte growth factor
TNF-α	Tumor Necrosis Factor-Alpha
SNAI1	Snail Homolog 1
SNAI2	Snail Homolog 2
TWIST1	Twist Homolog 1
SOX9	SRY (Sex Determining Region Y)-Box 9
ZEB1	Zinc Finger E-Box Binding Homeobox 1
CDH1	Cadherin 1, Type 1, E-Cadherin
CDH2	Cadherin 2, Type 1, N-Cadherin
VIM	Vimentin
HAS1	Hyaluronan Synthase 1
HAS2	Hyaluronan Synthase 2
TNC	Tenascin C
SPP1	Secreted Phosphoprotein 1
LUM	Lumican
SPARC	Secreted Protein, Acidic, Cysteine-Rich
POSTN	Periostin
COL6A3	Collagen, Type VI, Alpha 3
S100A4	S100 Calcium Binding Protein A4
SDC1	Syndecan 1
SMAD2	SMAD Family Member 2
SMAD3	SMAD Family Member 3
SMAD4	SMAD Family Member 4
WNT3A	Wingless-Type MMTV Integration Site Family, Member 3A
WNT5A	Wingless-Type MMTV Integration Site Family, Member 5A
CXCR2	Chemokine (C-X-C) Receptor 2
PPBP	Pro-Platelet Basic Protein (Chemokine (C-X-C) Ligand 7)
CXCR1	Chemokine (C-X-C) Receptor 1
JAG1	Jagged 1
GAPDH	Glyceraldehyde-3-Phosphate Dehydrogenase
GDC	Genomic DNA Control
RTC	Reverse Transcriptase Control
PPC	Positive PCR Control

*1.6. First-strand Complementary DNA Synthesis*

RNA was reverse transcribed to make complementary DNA (cDNA) using the RT^2^ First Strand kit (Qiagen, Germany) according to the manufacturer’s protocol. Each RNA sample’s genomic DNA elimination mix was briefly prepared by adding RNA (25ng-5μg), buffer GE (2ul), and RNase-free water to make the volume up to 10 μL. The mixture was incubated for 5 min at 42°C and immediately placed on ice for 1 min. Next, the reverse-transcription mix (20 μL) was prepared by adding 5× buffer BC3 (8 μL), Control P2 (2 μL), RE3 Reverse Transcriptase mix (4 μL), and RNase-free water (6 μL). Ten μL of reverse transcriptase mix was added to each tube containing 10 μL genomic DNA elimination mix and mixed gently by pipetting up and down. The mixture was incubated at 42°C for 15 min and the reaction immediately terminated by incubating at 95°C for 5 min. The volume of the mix was made to 111 μL by adding RNase-free water. The cDNA prepared was kept at -20°C till use.

**Table S3:** Custom PCR Array plate design and format

HIF1A 1	ARNT 2	EPAS1 3	TAZ 4	SIAH1 5	IL-6 6
IL-8 7	TGF-β1 8	VEGFA 9	EGF 10	FGF2 11	CXCL12 12
PDGFD 13	IGF2 14	BMI1 15	HGF 16	TNF-α 17	SNAI1 18
SNAI2 19	TWIST1 20	SOX9 21	ZEB1 22	CDH1 23	CDH2 24
VIM 25	HAS1 26	HAS2 27	TNC 28	SPP1 29	LUM 30
SPARC 31	POSTN 32	COL6A3 33	S100A4 34	SDC1 35	SMAD2 36
SMAD3 37	SMAD4 38	WNT3A 39	WNT5A 40	CXCR2 41	PPBP 42
CXCR1 43	JAG1 44	GAPDH 45	GDC 46	RTC 47	PPC 48

**Table S4A:** Gene symbols and their up-regulated expression (fold change) in stromal cells Hi-BCSCs_SC group (Test) as compared to Lo-BCSCs_SC group (Control)

Gene symbol	Fold regulation	Gene symbol	Fold regulation
HIF1A	2.61	TWIST1	3.61
ARNT	2.55	SOX9	3.4
EPAS1	2.36	CDH1	3.35
TAZ	2.56	VIM	5.33
IL-6	9.42	HAS2	2.21
IL-8	22.1	LUM	10.84
TGF-β1	2.03	SPARC	5.71
VEGFA	9.67	POSTN	3.35
FGF2	8.55	COL6A3	2.86
CXCL12	4.29	SMAD2	2.06
PDGFD	2.48	SMAD4	5.06
HGF	3.54	PPBP	9.65
TNF-α	6.01	JAG1	3.64

*1.7. Conventional GAPDH PCR for checking sample quality*

The RNA concentration and purity were assessed with Nanodrop 2000. Samples showing high concentrations and good quality were further processed for cDNA library construction. Before running the samples for RT-PCR, we performed conventional PCR for a housekeeping gene Glyceraldehyde 3-phosphate dehydrogenase (GAPDH). Samples showing good expression of GAPDH were processed further for gene expression analysis.

**Table S4B:** Gene symbols and their up-regulated expression (fold change) in cancer cells Hi-BCSCs_CC group (Test) as compared to Lo-BCSCs_CC group (Control)

Gene symbol	Fold regulation	Gene symbol	Fold regulation
IL-8	27.722	TWIST1	3.54
IL-6	22.18	ZEB1	3.08
LUM	20.36	HAS2	3.06
COL6A3	11.00	SMAD4	3.00
VIM	9.16	SIAH1	2.73
POSTN	8.28	SPARC	2.64
HIF1A	5.94	BMI1	2.55
CXCL12	5.83	PDGFD	2.51
SPP1	5.20	TNF-α	2.50
HGF	4.49	CDH2	2.44
VEGFA	4.45	ARNT	2.33
FGF2	3.68	TNC	2.26
S100A4	3.65	SMAD2	2.25

**Table S5:** Spearman’s rank correlation coefficients of differentially expressed genes (fold change) and BCSCs percentage

	Spearman’s rank correlation coefficient (ρ)	p-value
HIF1α	0.279	0.124
ARNT	0.313	0.096
EPAS1	0.068	0.390
TAZ	0.014	0.477
SIAH1	0.111	0.326
LUM	0.365	0.062
COL6A3	0.279	0.124
POSTN	0.361	0.064
SPP1	0.135	0.291
HAS2	0.235	0.166
SPARC	0.239	0.163
TNC	0.292	0.112
HGF	0.207	0.395
VEGFA*	0.552	0.014
FGF2	0.335	0.08
PDGFD	0.274	0.257
CXCL12*	0.453	0.026
PPBP	0.100	0.342
IL-6*	0.509	0.026

*1.8. Real-Time PCR*

For a 48-well array format, a total of 1350 μL of PCR mix was prepared by adding 675 μL 2X RT^2^ SYBR green master mix, 51 μL cDNA, and 624 μL RNase-free water. The reaction mixture was mixed well by gentle pipetting. 25 μL/well was dispensed into 48 wells (A1-A6, B1-B6, C1-C6, D1-D6, E1-E6, F1-F6, G1-G6, and H1-H6) of customized RT^2^ profiler PCR array. The plate was sealed carefully with optical adhesive film, centrifuged briefly, and placed in the real-time cycler programmed with conditions described in Table S6:

**Table S6:** Temperature conditions for real-time PCR array experiment

Program	Cycles	Duration	Temp.
Pre-incubation	1	10 min	95°C
Denaturation	45	15 s	95°C
Amplification	45	1 min	60°C
Melting curve analysis	1	5 s	95°C
Melting curve analysis	1	1 min	65°C
Melting curve analysis	1	-	95°C

The threshold cycle (Ct) for each well was calculated, and the data were analyzed using advanced online software RT^2^ Profiler PCR array data analysis version 3.5.
